# High throughput DNA sequencing to detect differences in the subgingival plaque microbiome in elderly subjects with and without dementia

**DOI:** 10.1186/2041-2223-3-19

**Published:** 2012-09-21

**Authors:** Andrew F Cockburn, Jonathan M Dehlin, Tiffany Ngan, Richard Crout, Goran Boskovic, James Denvir, Donald Primerano, Brenda L Plassman, Bei Wu, Christopher F Cuff

**Affiliations:** 1 Microbiology, Immunology & Cell Biology, School of Medicine, Robert C. Byrd Health Sciences Center, West Virginia University, P.O. Box 4622, Morgantown, WV, 26506-4622, USA; 2 Periodontics, School of Dentistry, Robert C. Byrd Health Sciences Center, West Virginia University, P.O. Box 9400, Morgantown, WV, 26506-9400, USA; 3 Department of Biochemistry and Microbiology, Robert C. Byrd Biotechnology Science Center, Joan C. Edwards School of Medicine, One John Marshall Drive, Huntington, WV, 25755, USA; 4 Department of Psychiatry and Behavioral Sciences, Duke University Medical Center, 2200 West Main Street, Durham, NC, 27706, USA; 5 School of Nursing and Global Health Institute, Duke University, 307 Trent Drive, Durham, NC, 27710, USA

**Keywords:** Cognitive impairment, Oral disease, Oral microbiome, Subgingival plaque

## Abstract

**Background:**

To investigate the potential association between oral health and cognitive function, a pilot study was conducted to evaluate high throughput DNA sequencing of the V3 region of the 16S ribosomal RNA gene for determining the relative abundance of bacterial taxa in subgingival plaque from older adults with or without dementia.

**Methods:**

Subgingival plaque samples were obtained from ten individuals at least 70 years old who participated in a study to assess oral health and cognitive function. DNA was isolated from the samples and a gene segment from the V3 portion of the 16S bacterial ribosomal RNA gene was amplified and sequenced using an Illumina HiSeq1000 DNA sequencer. Bacterial populations found in the subgingival plaque were identified and assessed with respect to the cognitive status and oral health of the participants who provided the samples.

**Results:**

More than two million high quality DNA sequences were obtained from each sample. Individuals differed greatly in the mix of phylotypes, but different sites from different subgingival depths in the same subject were usually similar. No consistent differences were observed in this small sample between subjects separated by levels of oral health, sex, or age; however a consistently higher level of Fusobacteriaceae and a generally lower level of Prevotellaceae was seen in subjects without dementia, although the difference did not reach statistical significance, possibly because of the small sample size.

**Conclusions:**

The results from this pilot study provide suggestive evidence that alterations in the subgingival microbiome are associated with changes in cognitive function, and provide support for an expanded analysis of the role of the oral microbiome in dementia.

## Background

In addition to the hypothesized link between oral health and chronic systemic diseases, such as cardiovascular disease, stroke, and diabetes, there now appears to be an association between oral health and neurodegenerative diseases, ranging from mild to moderate loss of cognitive function to Alzheimer’s Disease (AD) [1]. Poorer cognitive performance and tooth loss have been linked epidemiologically in both retrospective and prospective studies [2-7], and tooth loss has been associated with an increased risk of both dementia and cognitive decline [8]. Indeed, increasing tooth loss over time is associated with increased likelihood of low cognitive scores [2]. Beyond epidemiological associations, independent lines of experimental evidence support the hypothesis that bacteria associated with diseases of the oral cavity contribute to neurodegeneration. Oral bacteria and bacteria closely related to those found in the oral cavity have been found at a higher frequency *post mortem* in the brains of patients with AD than in those of patients who did not have AD [9,10]. In addition, the Third National Health and Nutrition Examination Survey (NHANES-III) provided evidence that gingival bleeding and loss of periodontal attachment were associated with lower cognitive function [11]. Furthermore, subjects with high levels of antibody against the periodontal pathogen *Porphyromonas gingivalis* had significantly greater impaired verbal memory and subtraction test performance, and this finding remained robust when adjusting for potential sociodemographic and vascular confounders [12]. Levels of immunoglobulin G (IgG) antibodies and serum tumor necrosis factor levels have been found to discriminate between normal subjects and AD patients [7], and recently Sparks Stein *et al*. [13] found that elevated antibody levels to periodontal disease bacteria were observed in subjects years before cognitive impairment, suggesting that periodontal disease could potentially contribute to the risk of AD onset or progression.

If oral health is linked to neurodegeneration, it is plausible that bacteria found in the oral cavity play a causal role in establishing this link. The collection of microorganisms in the oral cavity, the ‘oral microbiome’, has been studied using a variety of molecular methods that can identify both cultivatable and non-cultivatable bacteria within various ecological niches in the oral cavity [14,15]. Despite the evidence linking oral health and cognitive function, there is a paucity of empirical data that assess the oral microbiome in patients with cognitive degeneration [16]. Microbiome analysis could be used to determine whether the bacterial compositions of the oral microbiome or ‘bacterial signatures’ could serve as a predictive biomarker for increased risk of cognitive impairment. It is also possible that preventive treatment could target the makeup of the oral microbiome and the efficacy of such treatment could be monitored with this approach.

Recent advances in the availability and reduced costs of high throughput DNA sequencing and bioinformatics tools provide a broadly available and increasingly cost effective method to identify bacterial populations found in polymicrobial biofilms associated with human tissue, including the oral cavity [17-19]. The present study was undertaken to develop a sampling and analysis pipeline using next generation DNA sequencing technology that could be used to characterize microbial populations in subgingival plaque samples. Using an Illumina HiSeq1000 DNA sequencer and a sample preparation and analysis pipeline that enabled multiple samples to be sequenced within the same sequencing lane, we were able to generate and analyze economically more than one million bacterial DNA sequences from each of 15 subgingival plaque samples. The participants were enrolled in a study that assessed oral health and cognitive function among adults at least 70 years old from West Virginia, some of whom were from medically underserved communities [20]. These sequences were analyzed using bioinformatics tools available in the publicly accessable software package Quantitative Insights into Microbial Ecology (QIIME) [21], and sequence comparisons were made among participants who were clinically assessed as normal or exhibited alterations in cognitive function. The results provide a road map for future efforts to use high throughput DNA sequencing to characterize the oral microbiome in the context of systemic disease, and provide preliminary evidence that differences exist in the bacterial composition of subgingival plaque in patients with alterations in cognitive function. In contrast to other approaches to microbiome analysis, high throughput sequencing holds out the promise of also being useful for metagenomic analysis of the oral microbiome to identify potential virulence factors that contribute to systemic disease.

## Methods

### Oral health screening, cognitive analysis, and sample collection

All samples were collected under a protocol reviewed and approved by the West Virginia University Institutional Review Board. The criteria for study participants were age 70 years or older, resident of West Virginia, community-living, and at least four natural teeth. Oral evaluations were performed by calibrated researchers using guidelines from the NHANES 1999 to 2000 [22]. A psychometrician administered to the participants a battery of neuropsychological measures that assessed verbal and visual memory, language, executive function, orientation, praxis, and reading ability. Depression was assessed using the Geriatric Depression Scale [23]. A proxy informant, usually a spouse or adult child, provided information about the participant’s cognitive function, functional limitations, medical history, and medications. All collected data were reviewed by two study psychologists and diagnoses were assigned within three cognitive categories: normal cognitive function, cognitive impairment without dementia (CIND), and dementia. The Diagnostic and Statistical Manual of Mental Disorders, Fourth Edition, criteria were used for the diagnosis of dementia [24]. CIND was defined as mild cognitive or functional impairment reported by the participant or informant that did not meet the criteria for dementia, or performance on neuropsychological measures that was both below expectations based on reading ability and educational and occupational history, and at least 1.5 standard deviations below published norms on any test within a cognitive domain (for example, memory, orientation, language, executive function, praxis). Diagnoses were anchored by these criteria, but the final diagnoses were based on clinical judgment. Similar assessment and diagnostic procedures have been used and validated in multiple large epidemiological studies on cognitive impairment in later life [25,26].

A total of 15 samples of bacterial DNA from 10 individuals were sequenced for this report, four of which (N1, N2, C2, C3) were obtained and partially analyzed during an earlier phase of the study [16]. Eleven additional subgingival plaque samples were obtained using sterile periodontal curettes from pocket probing depths of 1 to 3 mm, 3 to 5 mm, or >5 mm. These samples were collected into tubes containing Invitek SalivaGene DNA stabilization buffer (STRATEC Molecular GmbH, Berlin, Germany). In most cases, multiple plaque samples from the same pocket probing depth in the same participant were pooled into one tube for DNA extraction.

### DNA extraction

DNA from the 11 new samples was purified using an Invitek PSP SalivaGene DNA Kit. As part of the purification procedure, 100 μg of lysozyme (Sigma-Aldrich, St. Louis, MO, USA) was added to each tube, the mixture incubated at 37°C for 10 minutes, and then processed according to the manufacturer's recommendations.

### PCR and fragment purification

PCR primers, conditions for amplification of sequences in the V3 region of the 16S ribosomal RNA gene, and a multiplexed DNA sequencing strategy were as described in Bartram *et al*. [27] unless otherwise indicated. The V3 region varies in length by about 30 base pairs among different species of bacteria in the Greengenes database, and the sequences obtained and analyzed in this study showed a similar size variability. The amplicon ranges from 296 to 327 base pairs, of which 160 base pairs is the primer. High pressure liquid chromatography-purified PCR primers were obtained from Integrated DNA Technologies (Coralville, IA, USA). Purified DNA was amplified using an AccuPrime PCR Kit (Invitrogen Life Technologies, Grand Island, NY, USA) on an MJ Research PTC-200 Thermal Cycler using the following conditions: 95°C for 6 minutes denature; 95°C for 2 minutes, 50°C for 2 minutes, 72°C for 2 minutes 30 cycles; 72°C for 4 minutes extend. Each reaction contained 0.5 μl TAQ polymerase, 5 μl 10x buffer 1(600 mM Tris-SO4 (pH 8.9), 180 mM (NH4)2SO4, 20 mM MgSO4, 2 mM dGTP, 2 mM dATP, 2 mM dTTP, 2 mM dCTP, thermostable AccuPrime™ protein, 10% glycerol), 20 μM forward primer, 20 μM reverse primer, and up to 60 ng DNA in a total volume of 50 μl. PCR reactions were performed in triplicate and reaction products were pooled prior to purification. Because there was a low concentration of DNA in some of the samples, it was necessary to perform 30 cycles of amplification to obtain sufficient material to view on a gel. Pooled PCR products were purified by electrophoresis through 2% agarose in Tris/borate/EDTA gels and the bands corresponding to approximately 300 base pairs were excised and purified using a QIAquick Gel Extraction Kit (Qiagen, Valencia, CA, USA) according to the manufacturer's directions.

### DNA sequencing

Indexed libraries were pooled so that 12 libraries were sequenced in each lane of the flow cell. Eight pmols of the pooled libraries were clustered onto an Illumina v2 sequencing flow cell using an Illumina cBOT. Libraries were then sequenced in a 2 x 125 bp paired-end strategy on an Illumina HiSeq1000, so that the forward and reverse reads could be assembled into a single contig. Reads were converted from Illumina bcl format to fastq format and separated into bins based on exact match to the index using CASAVA 1.8.2 (Illumina, San Diego CA, USA). An average of 6.7 million reads/sample passing the filter was sequenced in each library.

### DNA sequence processing

Sequence files were initially processed by removing sequences corresponding to linkers and primers by automated batch processing using scripts written in-house. In an effort to reduce artifacts generated by sequencing errors, a strict quality filtering protocol was employed that reduced the number of analyzed sequences to approximately 35% of the total number of sequences generated. Nevertheless, an average of more than two million high quality reads was obtained from each sample. Quality filtering of DNA sequences was performed using the following steps: 1) Sequences were first filtered by the Illumina software to eliminate the poorest reads (Q score ≥ 30) and imperfect primer matches. 2) The forward and reverse sequences were matched to construct a sequence that spanned the entire region between the primers with a program written in-house. The original Illumina sequences were all 125 bases in length, which is where the run ended. The pairing strategy overlaid the two 3’ ends starting with an overlap of 58 bases. The overlap window was extended one base at a time to 89 bases until a perfect match was obtained in the overlap region. Any pair of sequences that did not match at 100% identity in any of the size windows was discarded. This step eliminated 56% of the sequences, which overwhelmingly had the lowest quality scores. In general, the sequence quality was better in the middle than at the end, so this preferentially eliminated sequences with sequencing artifacts. 3) Paired sequences with a Phred quality score of less than five were discarded. This removed a few remaining low quality sequences, especially any that had low quality in the regions between the primers and the overlap. 4) The sequences were clustered by matching against the Greengenes database, which is a curated collection of known bacterial 16S sequences. Sequences that did not match any of the known bacterial sequences with 97% identity were discarded. This removed chimeras and most major PCR artifacts and represented approximately 5% of the total remaining sequences. Matching at 97% identity meant that any single base PCR artifacts would be combined with the corresponding authentic sequence (since the region is about 100 bases long, up to three single base changes will be ignored). 5) The resulting table of operational taxonomic units (OTUs) was filtered to remove any sequences that appeared less than 150 times.

Finally, scripts written in-house in biopython were used to convert the filtered Illumina data to the FASTA format for analysis by QIIME for taxonomic assignment and measurements of microbial diversity, but scripts to do this are now part of QIIME. To process Illumina-generated files in QIIME, the file headers were changed to begin with ‘>sample_number’ where ‘sample’ is the sample number and ‘number’ is the number of the sequence in the file. All of the sequences were then combined into one file for analysis with QIIME. DNA sequences generated and analyzed in this study can be found at the National Center for Biotechnology Information Sequence Read Archive, project number SRA057340.

### QIIME

All QIIME analyses were performed on a virtual server hosted by Amazon Web Services using an existing QIIME image. The server had the following specification: QIIME 1.4.0 EBS East XLARGE (ami-438d5b2a). The following QIIME scripts were used during analysis and default parameters were used unless otherwise noted: 1) ‘pick_reference_otus_through_otu_table.py’ matched sequences at 97% sequence identity with OTUs associated with specific bacterial phylotypes in the Greengenes database (4Feb2011); 2) ‘summarize_taxa_through_plots.py’ generated bar graphs of the relative abundance of different taxa in each sample; 3) ‘alpha_rarefaction.py’ generated alpha rarefication plots; 4) ‘pick_rep_set.py’, ‘align_seqs.py’, ‘filter_alignment.py’, and ‘make_phylogeny.py’ were chained to generate a phylogenetic tree of the OTUs; 5) ‘beta_diversity_through_plots.py’ (using the phylogenetic tree and the weighted UniFrac option) generated a beta diversity table and principle coordinate plots for the inter-subject diversity; and 6) ‘otu_category_significance.py’ generated analysis of variance (ANOVA) scores for all OTUs versus various categories. This script calculated raw, Bonferroni corrected, and false discovery rate corrected probabilities.

### Statistical analysis

Data analyses involved logistic regression as implemented in JMP/Pro Software (version 9.0.2) and random forests as implemented in R Software [28].

## Results

### Demographics and health status of study participants

A total of 15 samples was obtained from 10 individual participants. Participants ranged from 70 to 101 years old, were a nearly even mix of men and women, and all but one were self-identified as Caucasian. The number of teeth retained by the participants ranged from 7 to 22. Additional features of their oral health examinations are listed in Table 1.

**Table 1:** Demographics and health status of study participants

**Sample ID**	**Cognitive assessment**	**Age**	**Sex**	**Race**	**PPD**	**Number of teeth**	**Gingivitis score**	**Plaque Index**^**a**^	**Number of coronal caries**	**Number of root caries**
N1	Normal	77	m	w	2	22	0.18	0	0	1
N2	Normal	77	f	w	2	11	0.82	0	5	4
C1	CIND	>90	m	w	4	20	0.00	1	0	0
C2	CIND	>90	f	w	3	19	ND^b^	1	4	3
C3	CIND	74	f	b	3	22	0.73	0	0	0
D1	Dementia	82	m	w	4	07	0.14	0	0	0
D2	Dementia	78	f	w	4	28	1.00	1	0	0
D3	Dementia	89	f	w	4	14	0.71	2	13	3
D4	Dementia	70	m	w	6	19	1.00	3	36	0
D5	Dementia	>90	f	w	2	10	0.00	1	2	0

^a^As described in Methods; ^b^Not done. b, black; CIND, cognitively impaired, without dementia; f, female;,m, male; PPD, pocket probing depth (mm); w, white.

### Generation and filtering of DNA sequences

The Illumina sequencing run of the 15 DNA samples generated a total of more than 100 million paired-end DNA sequencing reads that met the initial quality filtering criteria (Table 2). Approximately 44 million reads could be aligned to produce a continuous sequence with at least a 58 base overlap with 100% identity and of these, more than 34 million DNA sequences passed all quality filtering steps. These sequences were clustered into OTUs matching the Greengenes database at 97% homology. An OTU clustered at 97% homology was considered a unique phylotype that approximates a ‘species’, but the 97% identity rule typically applies across the entire 16S rDNA sequence. If the V3 region is more or less variable this rule may not apply accurately to a shorter read length. An average of about 2.4 million sequences was analyzed per DNA sample. Approximately 34% of the initial output of DNA sequences could thus be assigned as high quality OTUs for analysis using this sequencing and quality filtering pipeline. OTUs containing fewer than 150 sequences were discarded, resulting in identification of a total of 492 OTUs in this study [See Additional file 1].

**Table 2:** Number of DNA sequences obtained during processing

**Processing step**	**Total**	**%**
Initial Number of Sequences	101,081,862	100
Successful end pairing	44,142,704	44
PHRED score >5	36,213,577	36
OTUs identified and analyzed	34,655,555	34
Average sequences/sample	2,310,370	n.a.

*n.a*. not applicable; *OTU* operational taxonomic units.

### Population diversity in samples

**Figure 1:**
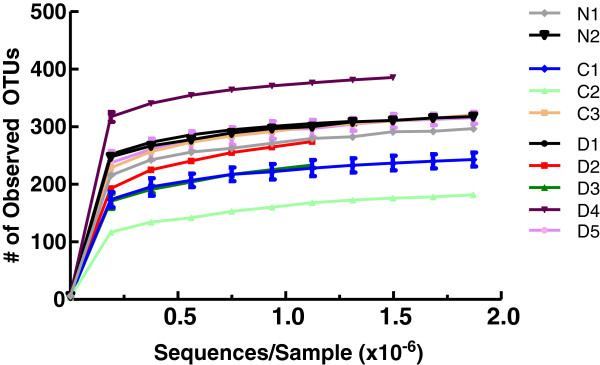
**Alpha rarefaction plot demonstrating phylotype diversity in subgingival plaque samples.** Shown are the numbers of different Operational Taxonomic Units (OTUs) as a function of the numbers of sequences analyzed and generated with QIIME. OTUs that occur less than 150 times/sample are not included. C, cognitively impaired, not dementia; D, dementia; N, normal.

Alpha diversity is the amount of population diversity within a given sample. Alpha diversity was measured as the number of phylotypes observed versus number of sequences analyzed. Once each sequence was assigned to an OTU, QIIME was used to assess the alpha diversity of each subject and generate diversification plots (Figure 1). The number of OTUs identified in subgingival plaque samples ranged from 182 (CIND2) to 385 (Dementia 4). Population diversity essentially plateaued after approximately 350,000 reads were analyzed, and this result was consistent across participants, irrespective of the final diversity. Using the QIIME script otu_category_significance.py to run ANOVA, the amount of diversity among participants was not linked to the method of DNA isolation, mental status, age, race, or parameters of oral health listed in Table 1 (data not shown). Although the population diversity varied among participants, multiple samples from individual participants yielded similar curves when analyzed separately. This characteristic is illustrated by the small error bars in Figure 1, where results from separate samples taken from different probing depths from some individuals were combined and averaged.

### Taxonomic assignments

**Figure 2:**
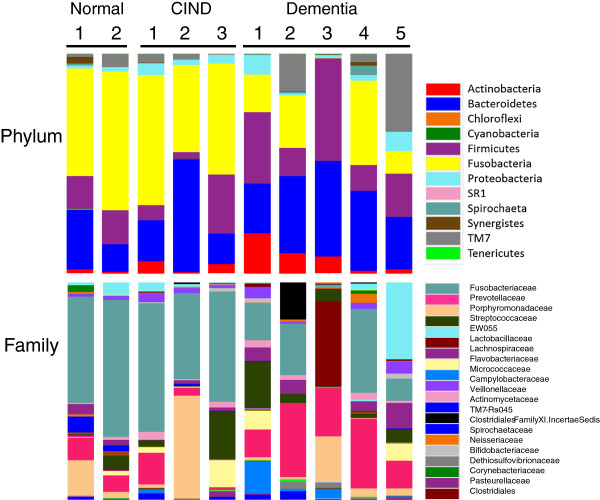
**Taxonomic assignments found in subgingival plaque clustered by cognitive status.** Counts for each OTU that was identified more than 150 times/sample were included in this analysis. The total height of the bar represents 100% of the assigned sequences after quality filtering, and the size of the colored regions represents proportional contributions of each phylotype shown. For clarity, only major families (>3%) are listed in the color key. OTU, Operational Taxonomic Units.

Taxonomic assignments for DNA sequences from each sample were made and analyzed at the phylum, class, order, family, and genus levels. Because of the relatively short region sequenced, assignments at the species level were not robust. Results are shown for analysis of samples at the phylum and family levels for each participant and for multiple probing depth sites where available. In general, most of the identified bacteria were distributed among the phyla Fusobacteria, Bacteroidetes, Firmicutes, TM7, Actinobacteria, and Proteobacteria with less than 1% contribution of bacteria from any other phyla (Figure 2). There appeared to be a higher proportion of Fusobacteria-specific sequences in the samples from participants who did not have dementia compared to those who did. Additionally, at the phylum level the proportion of sequences identified as members of the Bacteroidetes phylum seemed to be slightly elevated in the samples from subjects with dementia. These potential relationships were explored in more detail at lower taxonomic levels. Sixty-eight different families were identified during taxonomic assignment (Figure 2) and the large inter-person variation in bacterial populations became evident. Nevertheless, the most striking observation was that in a comparison of samples in the non-dementia versus the dementia groups, the non-dementia samples had a higher proportion of sequences identified as from the family Fusobacteriaceae (primarily genera *Fusobacterium* and *Leptotrichia*) and a lower proportion of sequences from Prevotellaceae (almost entirely *Prevotella*).

**Figure 3:**
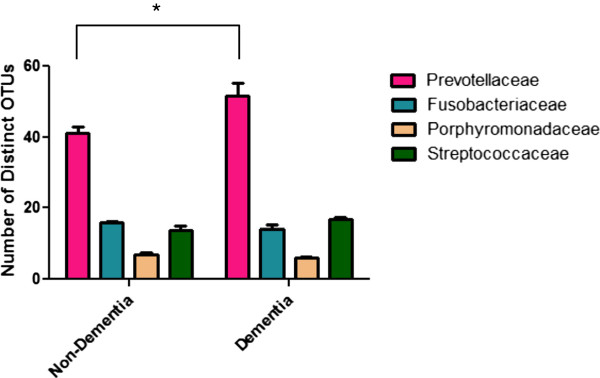
**High number of distinct OTUs assigned to Prevotellaceae from both dementia and non-dementia samples.** The number of distinct OTUs that are identified in four families that represent 2/3 of the total sequences were averaged for all samples and shown. Error bars represent standard error. Asterisk represents statistically significant difference by analysis of variance followed by Tukey’s multiple comparison test (*P* < 0.05). OTU, Operational Taxonomic Units.

The two most common families varied in the diversity of detected phylotypes. Sixty-nine phylotypes of *Prevotella* and one rare phylotype not assigned to a genus were seen in the Prevotellaceae. There was substantial diversity in the phylotypes of *Prevotella* (Figure 3), as has been recently reported in ethnically diverse populations including those of Aboriginal descent in Australia [29] and in a population from the Netherlands [19]. In contrast, only 17 phylotypes of Fusobacteriaceae were found, even though about twice as many sequences were assigned to this group. There were only five phylotypes of *Fusobacterium*, although this was the most common genus in the entire data set.

**Figure 4:**
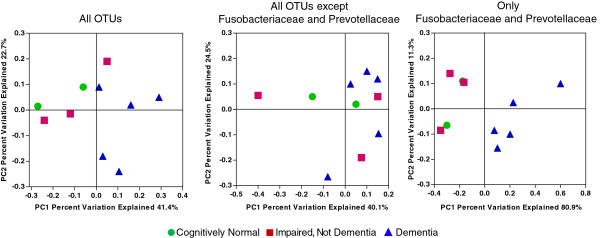
**Clustering of bacterial taxa by cognitive function.** Weighted UniFrac was used to generate a matrix of pairwise distances between communities and a scatterplot was generated from the matrix of distances using Principal Coordinate Analysis in QIIME. Each symbol represents the values of all samples from one participant analyzed collectively. In the left hand panel all OTUs that occur more than 150 times are included in the analysis. In the middle panel, the OTU table is further edited to remove any OTUs assigned to the Fusobacteriaceae or Prevotellaceae. In the right hand panel, only OTUs that were identified as Fusobacteriaceae or Prevotellaceae were analyzed. (green circle) cognitively normal, (red square) cognitively impaired without dementia, and (purple triangle) dementia. OTU, Operational Taxonomic Units.

Subroutines in QIIME were used to compute the UniFrac weighted beta diversity distance matrix among samples. Beta diversity is the measure of differences between samples in the abundance of phylotypes. Weighted UniFrac takes into account the phylogenetic distances between the OTUs, so that it captures differences not only at the level of individual phylotypes but also differences at higher taxonomic levels. The first two principal coordinates are plotted in Figure 4. The normal and CIND subjects tend to cluster in the top left of the graph and the subjects with dementia tend to be in the upper and lower right half of the graph. There is no obvious separation of normal from CIND. The first principal coordinate separates all but one of the normal/CIND subjects from the participants with dementia and the second principal coordinate provides some additional separation. The observed clustering in the non-dementia samples was due to differences in Fusobacteriaceae and Prevotellaceae. When the analysis was conducted with only Fusobacteriaceae and Prevotellaceae, the first coordinate completely separated the participants with dementia from the others. When all Fusobacteriaceae and Prevotellaceae were removed and the analyses repeated, there was no clustering of the non-dementia samples (Figure 4). Dementia versus non-dementia was the only variable that produced clustering on the principal coordinates plot. No clustering was evident for gingivitis score, age, sex, or number of teeth (data not shown). A variety of tests were used to determine whether these observed differences were statistically significant. Logistic regression analysis indicated that the levels of *Fusobacterium* and *Prevotella* were significantly different (*P* <0.0018 and *P* <0.0049, respectively) between non-dementia and dementia. However, random forest analysis, a somewhat more robust test of statistical significance indicated that the observed differences did not reach statistical significance.

**Figure 5:**
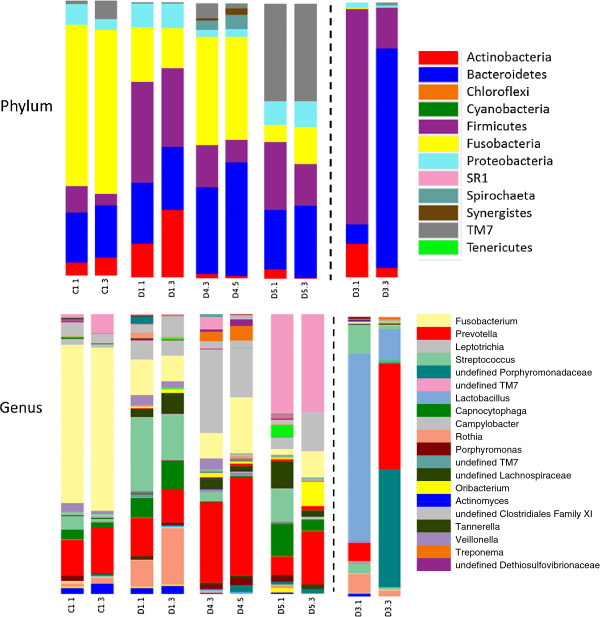
**Comparison of phylotypes found at various pocket probing depths (PPDs).** Sample identifications as in Table 1 and PPDs are listed at the base of each bar. For PPD: 1 = 1 to 3 mm, 3 = 3 to 5 mm 5 = > 5 mm. Results show at the phylum and genus levels. DNA samples obtained from various PPDs were sequenced and analyzed separately. The total height of the bar represents 100% of the assigned sequences after quality filtering, and the size of the colored regions represents proportional contributions of each phylotype shown. For clarity, only major genera (>3%) are listed in the color key.

Samples from multiple probing depths were obtained from 5 of the 10 participants and individual analysis of paired samples at the phylum and genus levels are shown in Figure 5. In samples from four of the five participants, subgingival plaque harbored similar distributions of genera irrespective of probing depth, although the percentages of minor genera differed substantially among participants. However, for the two samples from Dementia 3, the samples from the shallower probing depth of 3 to 5 mm contained high levels of lactobacilli and streptococci, whereas the sample from >5 mm contained a high level of undefined Porphyromonads.

## Discussion

The two purposes of this study were to develop a sample preparation and analysis pipeline to assess the oral microbiome using high throughput DNA sequencing, and to expand an ongoing study on the relationship between oral health and cognitive function in older West Virginians.

The major advantage of the Illumina platform is its capacity to generate millions of reads from each sample. Because of the relatively short read lengths, care must be used in choosing an appropriate region of the 16S RNA gene for analysis using the Illumina platform. The V3 region was selected because the primers used are the same as those used for older methods of bacterial community analysis, and this region had been used previously in Illumina-based analysis of microbial communities from environmental samples [27]. The region amplified in this study is longer (170 to 190 bases) than the V6 region (105 to 120 bases) [30] or the V5 region (approximately 82 bases) [18] sequenced in other studies. Using the PCR primers described in Bartram *et al*. [27] it was possible to run up to 12 samples per sequencing lane in this study, thereby substantially reducing the cost of the analysis. However, a challenge to using this system for microbiome analysis is the relatively short read lengths that are typically generated in a run (approximately 125 bp) and the lower quality of many of these reads. These disadvantages are obviated by using a paired-end sequencing approach, and successful microbiome analyses of various environmental niches [27] including the oral cavity [18,31] have been documented. Furthermore, recent additions to the QIIME program have streamlined analysis of Illumina-generated data. We used the Greengenes database to identify the taxa corresponding to our sequences. About 5% of our sequences were not found in Greengenes; we believe that most of these are artifacts, but it is possible that a small number of rare OTUs could have been excluded, which limits the utility of this approach for identifying very rare phylotypes with a high level of confidence. Nevertheless, we successfully obtained millions of sequences from each sample, yielding profound details of the structure of the microbiome in subgingival plaque.

Although the main goal of this pilot study was to work out methods for obtaining high quality data and performing subsequent analysis using validated, universally available software and databases, two interesting observations were made during the phylogenetic analysis of the data. First, a very high level of Fusobacteria was found, particularly in the samples from normal and CIND participants. Fusobacteria are well-studied anaerobes that have been found with great frequency in the oral cavity using culture-independent analyses [32-35], and members of the genus *Fusobacterium* were previously found to be among the most commonly identified species in the oral cavities of elderly patients [34,35], particularly in association with root caries [35]. A second novel observation was that the levels of Fusobacteriaceae were lower, and that levels of Prevotellaceae were higher in samples from subjects with dementia compared to subjects without dementia. We had hundreds of taxa in our results, so by chance some of them would likely appear to be correlated with dementia. However, Prevotellaceae and Fusobacteriaceae are the two most abundant families of bacteria, and antibody levels to individual species in those families have been shown to increase to higher levels in people who develop dementia than in those who do not [13].

There are four possible explanations for the correlations between dementia and components of the microbiome: 1) the correlations are spurious due to the small sample size; 2) dementia affects the microbiome; 3) the microbiome affects dementia; and 4) a third variable affects both.

First, we acknowledge that the sample size is small and that many more subjects need to be evaluated to obtain a robust result. Whether a larger sample size will confirm these preliminary observations is an open question.

Second, it might seem self-evident that individuals with dementia have poor oral hygiene resulting from changes in diet or oral hygiene behavior, and therefore worse oral health than individuals without dementia. As expected, the participants with dementia in this study had on average, slightly more gingivitis, fewer teeth, more caries, and much higher plaque indices. However, while this is true on average, it was not always the case on an individual basis. Participant Normal 2 had poor oral health while participants Dementia 1 and Dementia 5 had relatively good oral health, albeit with fewer teeth. Participant Dementia 2 had the highest number of teeth of all those in the study. If dementia causes poor oral health, which in turn causes the changes in the microbiome, then the correlations between the directly related parameters (cognition and oral health, or oral health and the microbiome) should be higher than the correlation between the indirectly related parameters (cognition and the microbiome). Since we found the opposite, the data do not support the hypothesis that the observed differences are merely secondary effects of poor oral hygiene in subjects with dementia.

We found more *Prevotella* on average in the samples from participants with dementia than in the samples from participants without dementia. However, the difference was not large and the statistical significance of that finding was dependent on the statistical test used to analyze the data. The number of Prevotellaceae phylotypes was high in both groups of samples, supporting many previous studies that showed diversity in Prevotellaceae phylotypes/species in the oral cavity [36]. In addition, there was a slight but statistically significant increase in the number of distinct OTUs in the dementia samples compared to the non-dementia samples, raising the question of whether there are phylotypes in the Prevotellaceae that contribute to dementia. At the species and strain levels there are examples of specific genes that could potentially contribute to virulence within the Prevotellaceae family including genes that encode fimbrial adhesins, phospholipases, host-resistance factors, adenine-specific DNA-methyltransferase and 8-amino-7-oxononanoate synthase [36,37]. Species-specific insertion sequences have also been identified [37], but whether these or other genes are disproportionately expressed in dementia patients and play a role in disease awaits metagenomic analyses. There were no other predominant phylotypes found in higher levels in participants with dementia compared to non-dementia, arguing against the idea that the presence of certain bacteria promotes dementia. However, the fact that higher levels of Fusobacteriaceae were found in all samples from participants without dementia suggests an alternate explanation, that perhaps certain oral bacteria provide protection against dementia, possibly by filling environmental niches that could be populated by more inflammatory microorganisms, by actively suppressing local or systemic inflammatory responses, or by producing biomolecules that are neuroprotective.

The final possibility is that both dementia and the microbiome are affected by a third variable. There is a strong genetic link to some forms of dementia, including the presence of the APOE-e4 variant of the Apolipoprotein E gene [38]. It is possible that the presence or absence of specific taxa could be due to genetic factors in the subject such as host immune responses, expression of adhesion molecules on host tissues that affect bacterial adherence, or other undefined factors. The relationship between human genotype and the oral microbiome needs to be studied carefully.

Sparks Stein *et al*. [13] found elevated levels of antibodies to *Prevotella intermedia* and *Fusobacterium nucleatum* in the blood of subjects who later developed AD. These investigators also found that subjects with Mild Cognitive Impairment (MCI), unlike AD subjects, had no differences in *P. intermedia* and *F. nucleatum* compared to normal subjects, but had reduced levels of antibodies to several other oral bacteria. Similarly, we found that our normal and CIND subjects did not separate based on their microbiome beta diversity and, in particular, that their Prevotellaceae and Fusobacteriaceae were similar. We hypothesize that our results can be reconciled with those of Sparks Stein *et al*. by predicting that subjects who will develop dementia have a leakier sub-gingival compartment resulting in increased interaction between the microbiome and the immune system, leading to higher antibody levels to the most prevalent bacteria: *Fusobacterium* and *Prevotella*. Compared to *Prevotella*, Fusobacteria are much less genetically diverse at the 16S gene, so they might be more sensitive to elevated serum antibody levels because of less diversity of surface proteins that could serve as targets for antibodies. Thus, later in life one might predict that higher levels of antibody might reduce levels of Fusobacteria yet fail to be as effective against genetically diverse *Prevotella*. Alternatively, it is possible that the difference in findings for Fusobacteriaceae might be because Sparks Stein *et al*. were using antibodies that would differentiate strains on the basis of surface proteins while we used 16S ribosomal sequences.

In summary, our results demonstrate, via high throughput DNA sequencing, that substantial inter-person variability exists in the oral microbiome of subgingival plaque. There appears to be a consistent difference in the levels of Fusobacteriaceae, and perhaps Prevotellaceae, in samples from patients who do or do not have dementia, which should be studied in more detail.

## Conclusions

We have shown that high throughput DNA sequencing is an effective and inexpensive method for analyzing the microbiome of oral subgingival plaque from individual subjects. It is sensitive enough to provide a measure of the bacteria from a single sampling site. Substantial inter-person variability exists in the sub-gingival plaque microbiome, while there is generally little variation at depths ranging from 1 to 5 mm in an individual subject's mouth. There appears to be a consistent difference in the levels of Fusobacteriaceae, and perhaps *Prevotella*, in samples from patients who do or do not have dementia, which should be studied in more detail.

## Abbreviations

AD: Alzheimer’s Disease; ANOVA: analysis of variance; bp: base pair; CIND: cognitive impairment without dementia; IgG: immunoglobulin G; MCI: Mild Cognitive Impairment; NHANES-III: Third National Health and Nutrition Examination Survey; OTU: operational taxonomic unit; PPD: pocket probing depth; QIIME: Quantitative Insights into Microbial Ecology.

## Competing interests

The authors declare that they have no competing interests.

## Authors’ contributions

AFC aided in study design, carried out DNA isolation, amplification, and purification, performed data analysis, and helped draft the manuscript. JD wrote programming code for aligning and organizing sequences and performed data analysis. TN coordinated the study, obtained samples, performed evaluations and organized clinical data. DP, GB, and JD participated in study design and performed DNA sequencing and primary evaluation of sequencing data. RC participated in study design, in recruitment and evaluation of participants and helped draft the manuscript. BW and BP participated in the design of the study and performed analysis of data related to cognitive function and helped draft the manuscript. CC conceived of the study, participated in its design and execution, and helped to draft the manuscript. All authors read and approved the final manuscript.

## Supplementary Material

Additional file 1 **Comma Separated Value (.csv).** Trimmed OTU Table Generated by QIIME. This is a comma separated value table that is generated by QIIME listing the number of hits for each OTU in each sample. The table has been trimmed to include all phylotypes that occur at least 150 times in the analysis. Click here for file
